# GLIM-Defined Malnutrition as a Predictor of Postoperative Morbidity and Survival After Curative Resection for Upper Gastrointestinal Cancer: A Systematic Review and Meta-Analysis

**DOI:** 10.3390/cancers18142217

**Published:** 2026-07-09

**Authors:** Ryota Matsui, Jun Watanabe, Kazuma Rifu, Kenichi Ishibayashi, Kenta Doden, Kengo Hayashi, Hiroto Saito, Megumi Watanabe, Toshikatsu Tsuji, Daisuke Yamamoto, Jun Kinoshita, Noriyuki Inaki

**Affiliations:** 1 Department of Gastrointestinal Surgery/Breast Surgery, Graduate School of Medical Science, Kanazawa University, 13-1 Takara-Machi, Kanazawa 920-8641, Ishikawa, Japanjunkino@staff.kanazawa-u.ac.jp (J.K.);; 2 Department of Surgery, Division of Gastroenterological, General and Transplant Surgery, Jichi Medical University, 3311-1 Yakushiji, Shimotsuke 329-0498, Tochigi, Japan

**Keywords:** gastrointestinal cancer, malnutrition, overall survival, postoperative complication, prognosis

## Abstract

**Background**: Malnutrition is common among patients with cancer and has been associated with unfavorable postoperative outcomes, including higher rates of complications. This study aimed to synthesize current evidence on the relationship between malnutrition, as defined by the Global Leadership Initiative on Malnutrition (GLIM) criteria, and postoperative outcomes after curative surgery for upper gastrointestinal (GI) cancers. **Methods:** We systematically searched the literature from database inception to 28 March 2026. Studies were eligible if they included adult patients with upper GI cancer who underwent surgical resection and had their nutritional status evaluated according to the GLIM criteria. We conducted a systematic review and random-effects meta-analysis. The primary outcomes were overall survival and overall postoperative complications, defined as events with a Clavien–Dindo grade ≥ II occurring within 30 days after surgery. Hazard ratios with 95% confidence intervals for overall survival and risk ratios with 95% CIs for postoperative complications were pooled. **Results:** Fourteen studies (17 reports) including a total of 7876 patients were eligible for both qualitative and quantitative synthesis. Compared with patients without malnutrition, those with GLIM-defined malnutrition showed poorer overall survival (hazard ratio: 1.96, 95% confidence interval: 1.57–2.45) and a higher risk of postoperative complications (risk ratio: 1.43, 95% confidence interval: 1.14–1.79). **Conclusions:** GLIM-defined malnutrition was associated with shorter overall survival and a higher incidence of postoperative complications in patients with upper GI cancer after surgery. However, because most included studies were observational and at low-to-moderate certainty of evidence, these associations should be interpreted with appropriate caution.

## Simple Summary

There have been no globally standardized diagnostic criteria for malnutrition. In 2019, diagnostic criteria for malnutrition that met global consensus were published, and standardized nutritional assessment is now required worldwide. This study demonstrated the impact of malnutrition, as defined by these standardized criteria, on postoperative outcomes for upper gastrointestinal cancer. GLIM-defined malnutrition was associated with almost a twofold increase in the hazard of death (hazard ratio: 1.96, 95% confidence interval: 1.57–2.45), as well as higher risks of severe and infectious complications, anastomotic leakage, postoperative pneumonia, and prolonged hospital stay. It is expected that these malnutrition criteria will serve as the basis for preoperative nutritional interventions in the future.

## 1. Introduction

Malnutrition is known to adversely affect surgical outcomes in patients with cancer, being linked to higher rates of postoperative complications and poorer long-term prognosis [1,2]. The detrimental impact of malnutrition on surgical outcomes has been recognized for decades, with early experimental and clinical work in the mid-20th century demonstrating that protein–energy deficits impair wound healing, increase postoperative complications, and worsen survival in surgical patients. Building on this long-standing foundation, recent initiatives have focused on standardizing how malnutrition is defined and operationalized in contemporary clinical research. To reduce these adverse consequences, current guidelines recommend 7–10 days of nutritional conditioning for mildly malnourished patients and 10–14 days for those with severe malnutrition before surgery [1,2]. Several international guidelines, including those from ESPEN and other professional societies, provide detailed recommendations for energy and protein targets, preferred routes of nutritional support, and suggested durations of preoperative conditioning in malnourished surgical patients. However, these recommendations are largely based on heterogeneous definitions of malnutrition and on mixed cancer or non-cancer populations.

The Global Leadership Initiative on Malnutrition (GLIM) criteria, established in 2019 as an international consensus definition of malnutrition [3], do not introduce a new biological concept of malnutrition but rather provide a unified framework for identifying and grading malnutrition based on phenotypic (body weight loss (BWL), low body mass index (BMI), reduced muscle mass) and etiologic (reduced intake or inflammation) criteria. Because approaches to diagnosing malnutrition and selecting candidates for preoperative nutritional support vary across countries and institutions, standardizing the diagnostic framework for malnutrition is essential. This standardization is intended to facilitate consistent nutritional assessment, allow comparability across studies, and support the design of GLIM-based interventional trials.

The association between malnutrition defined by the GLIM criteria and postoperative outcomes in patients with upper gastrointestinal (GI) cancer has not been fully clarified, largely because only a limited number of studies are available. A previous systematic review identified few prospective investigations focusing on GLIM-defined malnutrition [4]. In addition, the impact of malnutrition severity on postoperative outcomes remains insufficiently explored. Under the GLIM framework, the severity of malnutrition is graded using skeletal muscle mass, BMI, and the rate of BWL. Postoperative complications have been associated with a BWL of more than 10% and with reduced skeletal muscle mass [5,6,7,8]. If this is the case, patients with severe malnutrition would be expected to experience worse postoperative outcomes than those with moderate malnutrition. However, it is still unclear how postoperative results vary according to the severity of malnutrition as defined by the GLIM criteria.

We aimed to synthesize the emerging evidence specifically using the GLIM criteria in patients with upper GI cancer undergoing curative resection, thereby quantifying the impact of GLIM-defined malnutrition on postoperative outcomes and assessing how strongly this consensus definition captures the well-known risk associated with poor nutritional status in this population. Based on existing evidence, our hypothesis was that increasing malnutrition severity would be associated with a higher risk of postoperative complications and poorer long-term outcomes, such as survival and recurrence.

## 2. Materials and Methods

This study was designed and reported in accordance with the Preferred Reporting Items for Systematic Reviews and Meta-Analyses 2020 (PRISMA 2020; Supplementary S1) guidelines [9]. The review protocol was registered in the OSF international database (registration DOI: 10.17605/OSF.IO/VYSWT).

### 2.1. Including or Excluding Criteria

We included observational studies of adults (≥18 years) with upper GI cancer who underwent surgical treatment and had their nutritional status assessed using the GLIM criteria. The GLIM criteria require at least one phenotypic and one etiologic criterion to establish a diagnosis of malnutrition. In line with the original GLIM consensus, we considered unintentional weight loss, low BMI, and reduced muscle mass as phenotypic criteria, and reduced food intake and inflammation as etiologic criteria. A summary of the GLIM diagnostic framework and the cut off values used in the included studies is provided in Table 1.

**Figure 1:**
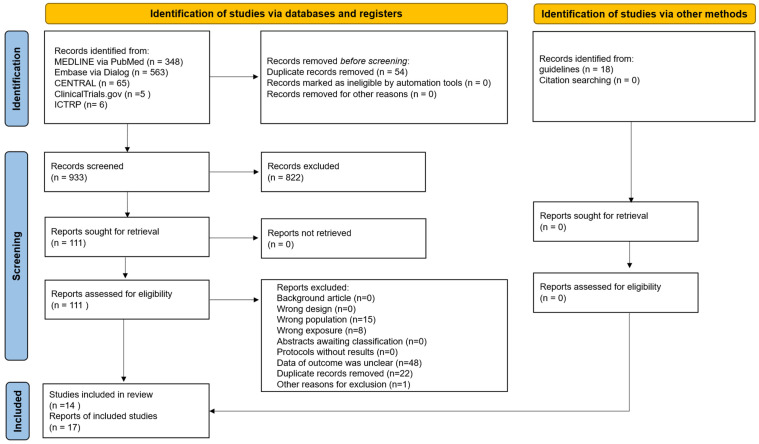
PRISMA 2020 flow diagram.

We excluded records for the following reasons: (1) ineligible study design (e.g., reviews, editorials, case reports, conference abstracts without sufficient data); (2) ineligible population (non-upper GI cancer, benign disease, or mixed populations without separable data); (3) no GLIM-based nutritional assessment; (4) no relevant postoperative or survival outcomes; (5) duplicate or clearly overlapping reports; and (6) other reasons (e.g., non-human studies, irretrievable full text). The numbers corresponding to each exclusion category are summarized in Figure 1. We identified eligible reports published up to 28 March 2026. In this review, GLIM-defined malnutrition was treated as the exposure, and the control group comprised patients with upper GI cancer who were not malnourished according to GLIM criteria. All studies meeting these criteria were included in the qualitative synthesis, whereas only those providing relevant postoperative or long-term outcome data were entered into the quantitative meta-analyses.

We searched for both published and unpublished studies in major electronic databases, including the Cochrane Central Register of Controlled Trials (Cochrane Library), MEDLINE (PubMed), and EMBASE (Dialog) (Supplementary S2). We also screened trial registries via the World Health Organization International Clinical Trials Registry Platform (ICTRP) and ClinicalTrials.gov (Supplementary S3). In addition, we examined reference lists of included studies, relevant international guidelines and standards [10,11,12,13,14,15,16,17,18,19,20,21,22,23,24,25,26,27], and articles that cited key studies to identify any further eligible trials.

### 2.2. Definition of Outcomes

The primary outcomes were overall survival (OS) and postoperative complications, defined as events of Clavien–Dindo (CD) grade ≥ II occurring within 30 days after surgery. Secondary outcomes were postoperative length of hospital stay and relapse-free survival (RFS). In a predefined sub-analysis of postoperative complications, we further evaluated the incidence of anastomotic leakage, postoperative pneumonia, infectious complications, severe complications classified as CD grade ≥ III, and postoperative mortality.

### 2.3. Data Extraction and Quality Assessment

Two independent reviewers first screened titles and abstracts, then assessed the full texts to determine study eligibility. After data extraction, the same reviewers independently evaluated risk of bias using the Quality In Prognosis Studies (QUIPS) tool and graded the certainty of evidence (COE) according to the Grading of Recommendations, Assessment, Development, and Evaluation (GRADE) approach [28]. Any disagreements between the two reviewers were resolved through discussion, and if consensus could not be reached, a third reviewer adjudicated. When essential methodological or outcome data were missing, we contacted the original study authors to obtain additional information.

### 2.4. Statistical Analysis

We calculated pooled effect estimates by summarizing hazard ratios (HRs) with 95% confidence intervals (CIs) for OS and RFS, mean differences (MDs) with 95% CIs for postoperative length of hospital stay, and risk ratios (RRs) with 95% CIs for postoperative complications and postoperative mortality. For dichotomous outcomes, we used an intention-to-treat principle whenever possible. Statistical heterogeneity was assessed using the I^2^ statistic together with visual inspection of forest plots, and potential sources of substantial heterogeneity (I^2^ > 50%) were explored [29]. Reporting bias was screened by checking major clinical trial registries (ClinicalTrials.gov and ICTRP), and when at least 10 studies were available for a given endpoint, potential publication bias was evaluated by visual inspection of funnel plots, following recommendations in the Cochrane Handbook [29].

We performed all meta-analyses using a random-effects model implemented in STATA SE16 (version 16.1, StataCorp, College Station, TX, USA) and Review Manager 5.4.2. In line with Cochrane Handbook recommendations [29], we assumed that the true intervention effects would vary across studies due to clinical heterogeneity and unmeasured patient-level factors, and therefore used the random-effects framework rather than a fixed-effect model. We rated the COE for each outcome using the GRADE framework, summarizing the overall confidence in the prognostic value of GLIM-defined malnutrition [29]. For this prognostic review, we initially classified observational evidence as high COE and then downgraded the rating in any domain where we identified serious concerns, such as risk of bias, inconsistency, indirectness, imprecision, or publication bias [29].

When sufficient data were available to explore potential effect modifiers, we performed predefined subgroup analyses according to cancer type (esophageal or gastric), malnutrition severity (moderate or severe), surgical approach (laparoscopic or open), and the method used to assess skeletal muscle mass (computed tomography (CT), magnetic resonance imaging (MRI), bioelectrical impedance analysis (BIA), dual-energy X-ray absorptiometry (DXA), or calf circumference). In analyses stratified by surgical approach, we categorized each study according to the technique most commonly used within that cohort. We also conducted sensitivity analyses excluding studies that did not directly measure muscle mass to assess the robustness of our findings.

## 3. Results

### 3.1. Study Selection

Figure 1 depicts the PRISMA study selection flowchart. Between database inception and March 28, 2026, we identified 933 records through electronic and registry searches. After screening and eligibility assessment, 14 studies (reported in 17 articles) comprising 7876 patients were included in both the qualitative and quantitative syntheses [30,31,32,33,34,35,36,37,38,39,40,41,42,43,44,45,46]. Although we followed Cochrane Handbook recommendations to incorporate unpublished and ongoing studies, no additional eligible records were retrieved from trial registries. As detailed in Figure 1, the main reasons for exclusion were insufficient outcome data (n = 48), incorrect exposure definition (n = 8), ineligible population (n = 15), and other reasons (n = 1).

### 3.2. Study and Participant Characteristics

**Figure 2:**
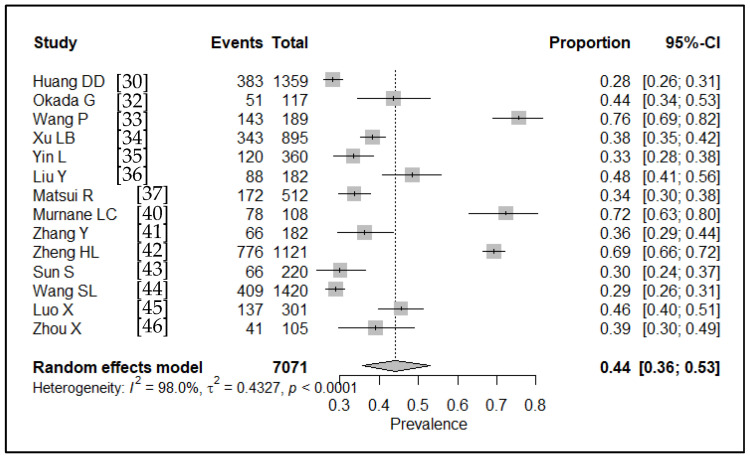
Prevalence of GLIM-defined malnutrition.

Table 2 summarizes the main characteristics of the 14 studies included in the quantitative synthesis. Three studies were designed as prospective cohort studies, whereas the remaining 11 were retrospective cohort studies. Nine studies enrolled patients with gastric cancer, four focused on esophageal cancer, and one included a mixed population with gastric and esophageal cancers. Skeletal muscle mass was assessed by computed tomography in nine studies, by anthropometric measurements in two studies, and by bioelectrical impedance analysis in two studies, while one study did not evaluate muscle mass. Across all included cohorts, the pooled prevalence of GLIM-defined malnutrition was 44.0% (95% CI: 36.0–53.0%), as shown in Figure 2.

### 3.3. Risk of Bias

The risk of bias assessment for the included studies is summarized in Supplementary S4. For OS, the risk of bias was generally low or high for outcome measurement, and low to moderate for statistical analysis, assessment of the prognostic factor, and study participation. In contrast, the domains of confounding and study attrition frequently showed moderate to high risk of bias, reflecting limitations in adjustment for prognostic covariates and loss to follow-up. For postoperative complications, the risk of bias for prognostic factor measurement was mostly low to moderate, while study confounding and statistical analysis were often at moderate or high risk of bias. By comparison, study participation, study attrition, and outcome measurement for postoperative complications were typically judged to be at low risk of bias.

### 3.4. Results of Meta-Analysis

Table 3 summarizes the certainty of evidence for each outcome as assessed using the GRADE approach. Survival outcomes were not reported separately as cancer-specific or non-cancer causes of death in the included studies. Because all studies analyzed outcomes using the original GLIM-defined malnutrition categories and none relied on imputed data, we were unable to conduct the planned sensitivity analyses based on different definitions or handling of missing data.

#### 3.4.1. Overall Survival

**Figure 3:**
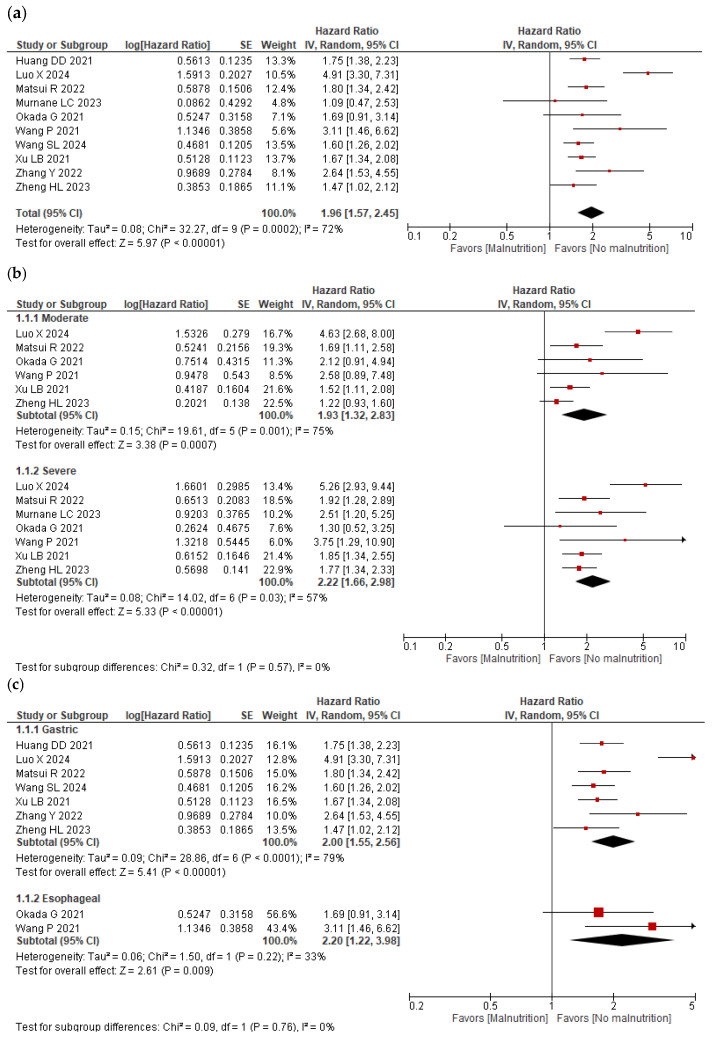

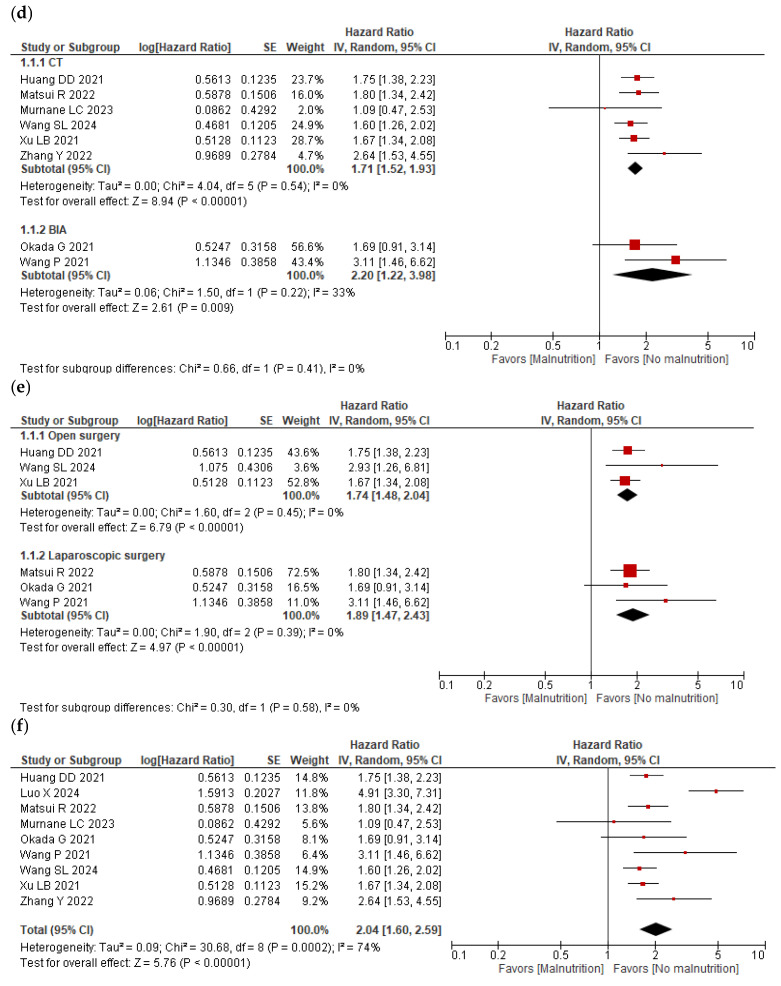
Results of meta-analyses: (**a**) overall survival; (**b**) subgroup analysis by malnutrition severity; (**c**) subgroup analysis by cancer type; (**d**) subgroup analysis by method used to assess skeletal muscle mass; (**e**) subgroup analysis by surgical approach; and (**f**) sensitivity analysis excluding studies without skeletal muscle mass assessment.

OS was reported in 10 studies. GLIM-defined malnutrition was associated with worse OS (HR: 1.96, 95% CI: 1.57–2.45, I^2^ = 72%, n = 10, COE: low; Figure 3a). Subgroup analyses showed no clear differences in this association when stratified by malnutrition severity (Figure 3b), cancer type (Figure 3c), method of skeletal muscle mass assessment (Figure 3d), or surgical approach (Figure 3e), although these comparisons were limited by the small number of studies and events. No additional subgroup analyses were undertaken because of insufficient data. Sensitivity analysis excluding studies without direct muscle mass assessment yielded effect estimates (HR: 2.04, 95% CI: 1.60–2.59; Figure 3f) that were broadly consistent with the primary analysis, supporting the robustness of the association between GLIM-defined malnutrition and poorer OS.

#### 3.4.2. Relapse-Free Survival

**Figure 4:**
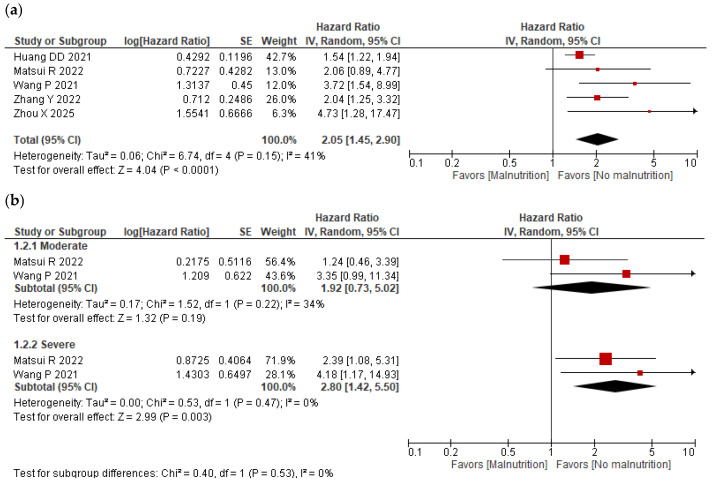
Results of meta-analyses: (**a**) relapse-free survival; (**b**) subgroup analysis according to malnutrition severity.

RFS was reported in five studies. GLIM-defined malnutrition was associated with poorer RFS (HR: 2.05, 95% CI: 1.45–2.90, I^2^ = 41%, n = 5, COE: moderate; Figure 4a). Subgroup analysis by malnutrition severity did not show meaningful differences between categories (Figure 4b). Because of the limited number of studies and events, no additional subgroup or sensitivity analyses were conducted for RFS.

#### 3.4.3. Total Postoperative Complications

**Figure 5:**
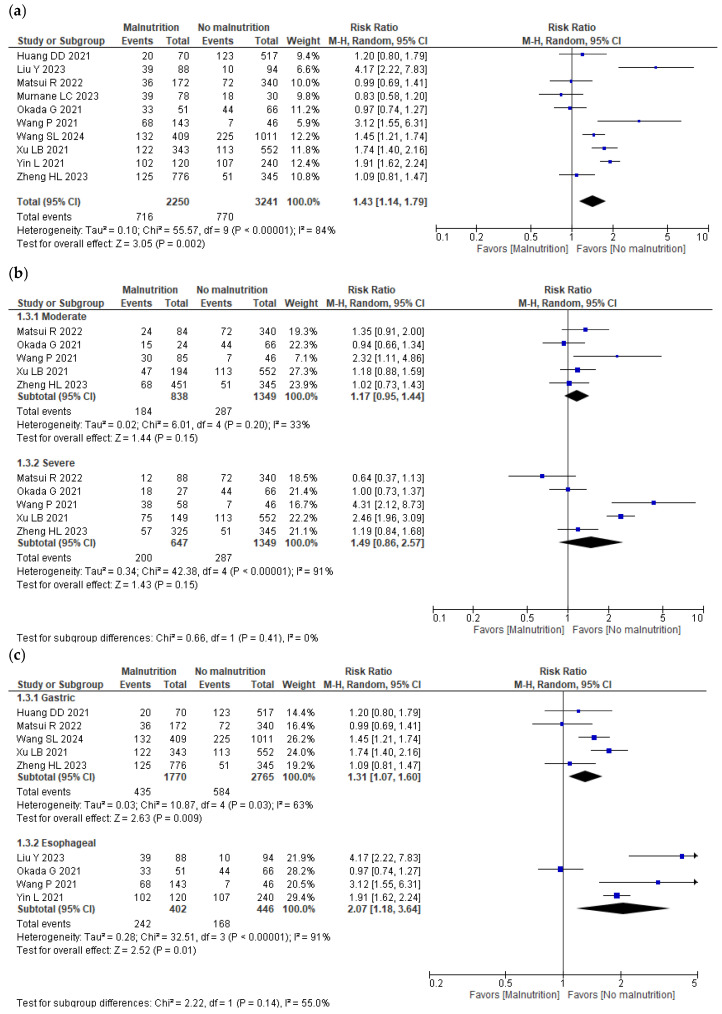

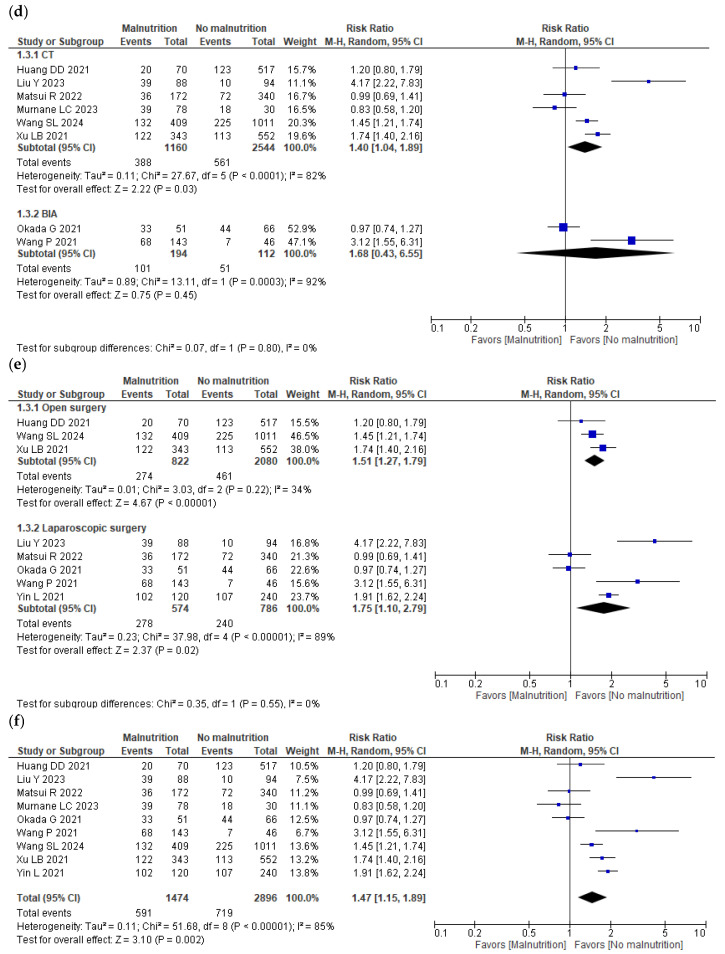
Results of meta-analyses: (**a**) total postoperative complications; (**b**) subgroup analysis according to malnutrition severity; (**c**) subgroup analysis by cancer type; (**d**) subgroup analysis according to method used to assess skeletal muscle mass; (**e**) subgroup analysis according to surgical approach; and (**f**) sensitivity analysis excluding studies without skeletal muscle mass assessment.

Postoperative complications were reported in 10 studies. GLIM-defined malnutrition was associated with an increased risk of overall complications (RR: 1.43, 95% CI: 1.14–1.79, I^2^ = 84%, n = 10, COE: low; Figure 5a). Subgroup analyses did not show clear differences in this association when stratified by malnutrition severity (Figure 5b), cancer type (Figure 5c), method used to assess skeletal muscle mass (Figure 5d), or surgical approach (Figure 5e), although these comparisons were limited by the small number of studies in each subgroup. No additional subgroup analyses were undertaken because of insufficient data. A sensitivity analysis excluding studies that did not evaluate skeletal muscle mass yielded similar estimates (RR: 1.47, 95% CI: 1.15–1.89; Figure 5f), supporting the robustness of the association between GLIM-defined malnutrition and higher postoperative complication rates.

#### 3.4.4. Severe Complications

**Figure 6:**
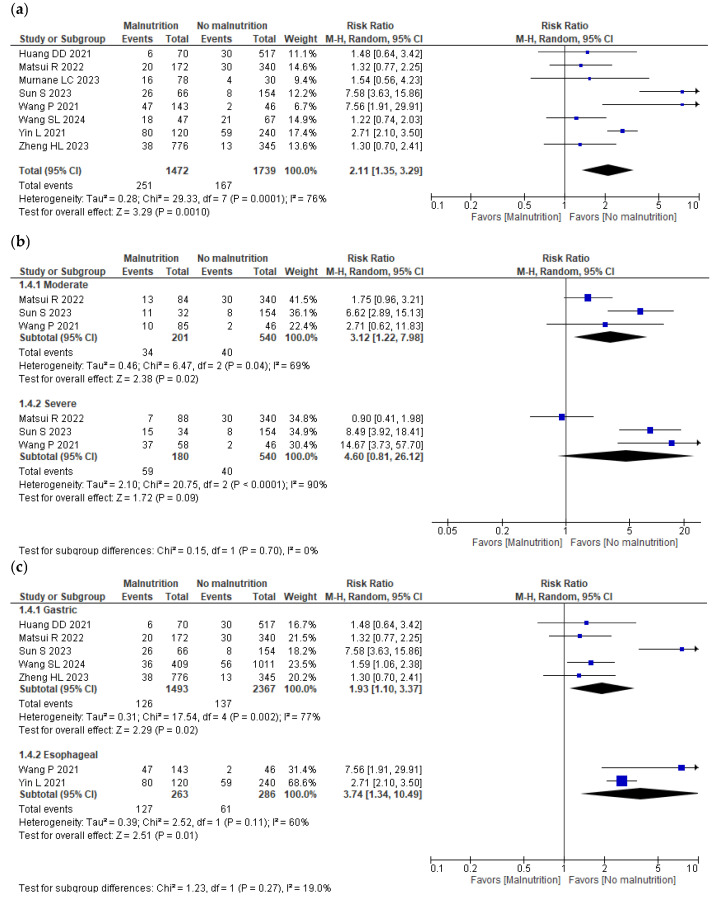

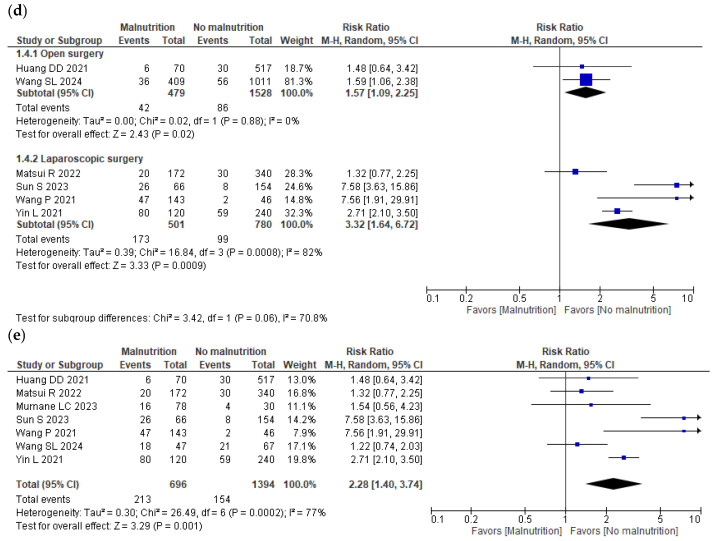
Results of meta-analyses: (**a**) severe complications; (**b**) subgroup analysis according to malnutrition severity; (**c**) subgroup analysis by cancer type; (**d**) subgroup analysis according to surgical approach; and (**e**) sensitivity analysis excluding studies without skeletal muscle mass assessment.

Severe postoperative complications were reported in eight studies. GLIM-defined malnutrition was associated with a higher risk of severe complications (RR: 2.11, 95% CI: 1.35–3.29, I^2^ = 76%, n = 8, COE: low; Figure 6a). Subgroup analyses showed no clear differences in this association when stratified by malnutrition severity (Figure 6b), cancer type (Figure 6c), or surgical approach (Figure 6d), although interpretation was limited by the small number of studies within each subgroup. Because of limited data, no additional subgroup analyses were conducted. In a sensitivity analysis excluding studies that did not assess skeletal muscle mass, patients with GLIM-defined malnutrition continued to show an increased risk of severe complications (RR: 2.28, 95% CI: 1.40–3.74; Figure 6e), supporting the robustness of this finding.

#### 3.4.5. Infectious Complications

**Figure 7:**
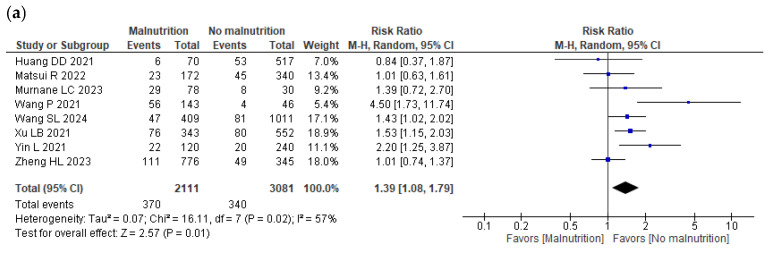

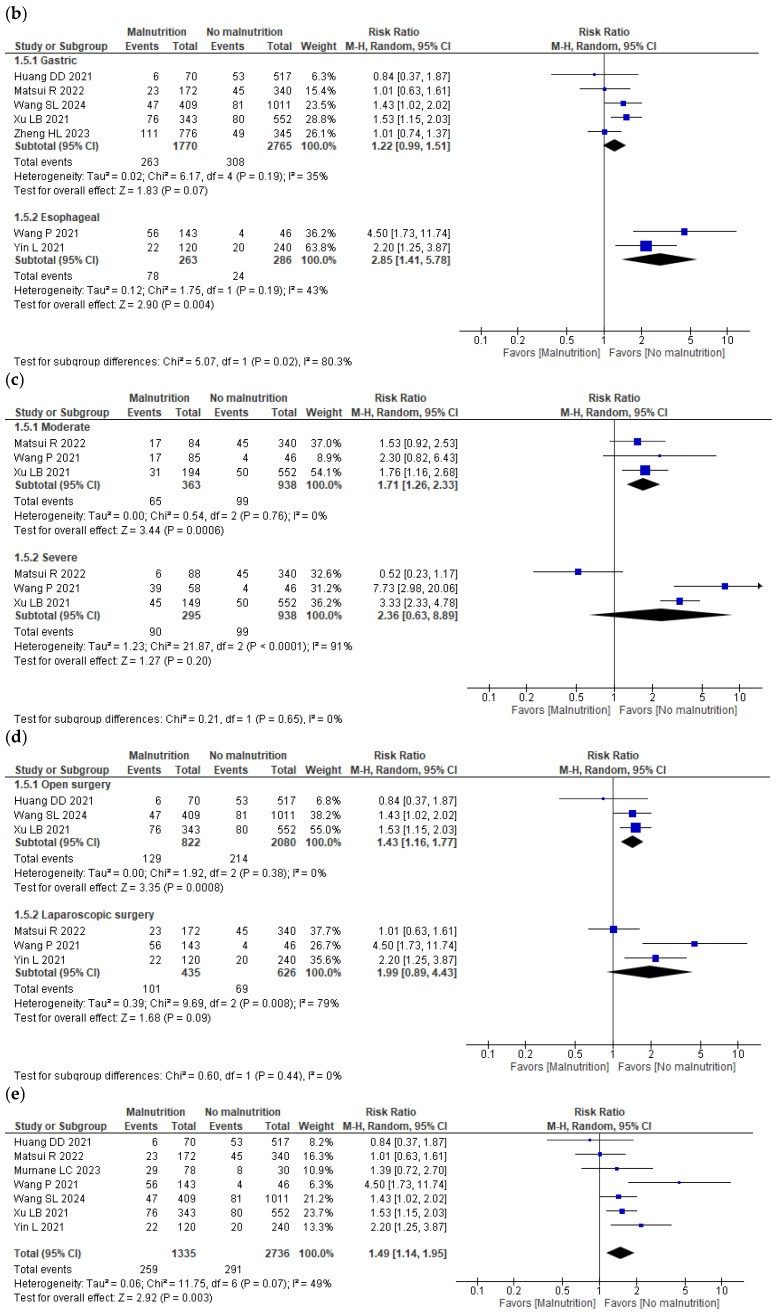
Results of meta-analyses: (**a**) infectious complications; (**b**) subgroup analysis by cancer type; (**c**) subgroup analysis according to malnutrition severity; (**d**) subgroup analysis according to surgical approach; and (**e**) sensitivity analysis excluding studies without skeletal muscle mass assessment.

Infectious complications were reported in eight studies. GLIM-defined malnutrition was associated with a higher risk of infectious complications (RR: 1.39, 95% CI: 1.08–1.79, I^2^ = 57%, n = 8, COE: moderate; Figure 7a). Subgroup analysis by cancer type indicated a significantly greater increase in infectious complications among patients with esophageal cancer compared with those with gastric cancer (P = 0.02; Figure 7b). In contrast, no clear differences were observed when stratifying by malnutrition severity (Figure 7c) or surgical approach (Figure 7d). Due to the limited number of available studies, no additional subgroup analyses were undertaken. Sensitivity analysis excluding studies that did not assess skeletal muscle mass produced similar effect estimates (RR: 1.49, 95% CI: 1.14–1.95; Figure 7e), supporting the robustness of the association between GLIM-defined malnutrition and increased infectious complications.

#### 3.4.6. Anastomotic Leakage

**Figure 8:**
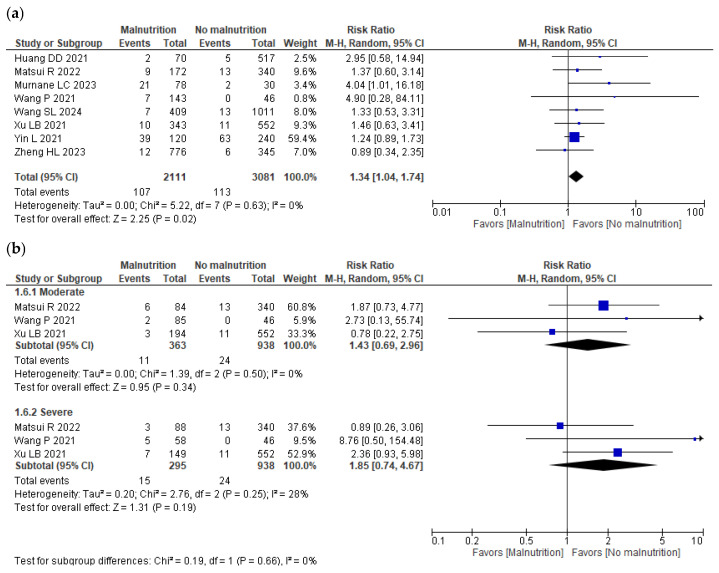

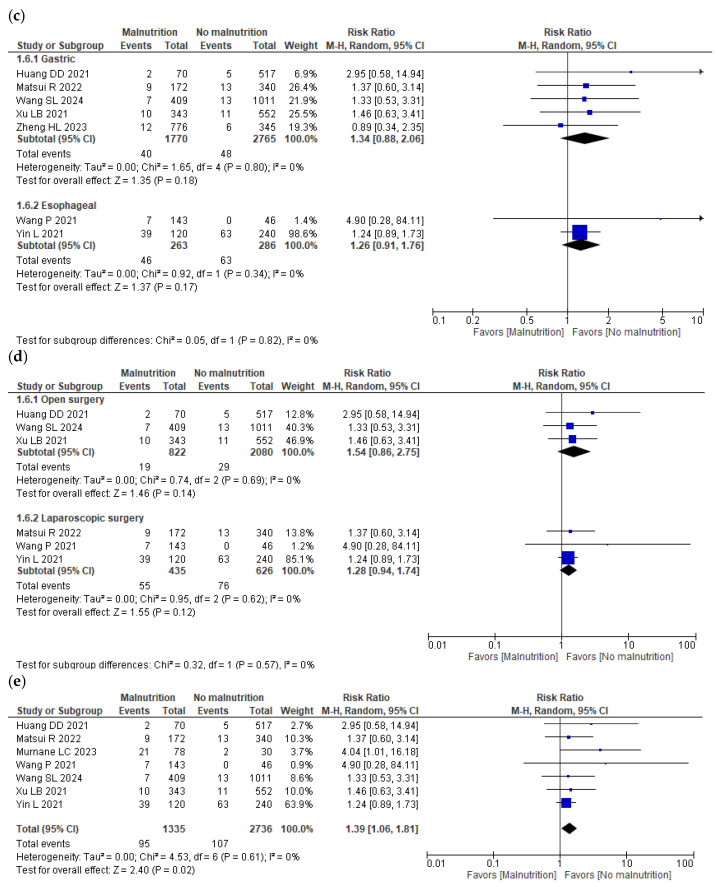
Results of meta-analyses: (**a**) anastomotic leakage; (**b**) subgroup analysis according to malnutrition severity; (**c**) subgroup analysis by cancer type; (**d**) subgroup analysis according to surgical approach; and (**e**) sensitivity analysis excluding studies without skeletal muscle mass assessment.

Anastomotic leakage was reported in eight studies. GLIM-defined malnutrition was associated with an increased risk of anastomotic leakage (RR: 1.34, 95% CI: 1.04–1.74, I^2^ = 0%, n = 8, COE: moderate; Figure 8a). Subgroup analyses showed no clear differences in this association when stratified by malnutrition severity (Figure 8b), cancer type (Figure 8c), or surgical approach (Figure 8d), although interpretation was limited by the small number of studies in each subgroup. Because of limited data, no additional subgroup analyses were conducted. A sensitivity analysis excluding studies that did not assess skeletal muscle mass yielded similar effect estimates (RR: 1.39, 95% CI: 1.06–1.81; Figure 8e), supporting the robustness of the association between GLIM-defined malnutrition and a higher risk of anastomotic leakage.

#### 3.4.7. Postoperative Pneumonia

**Figure 9:**
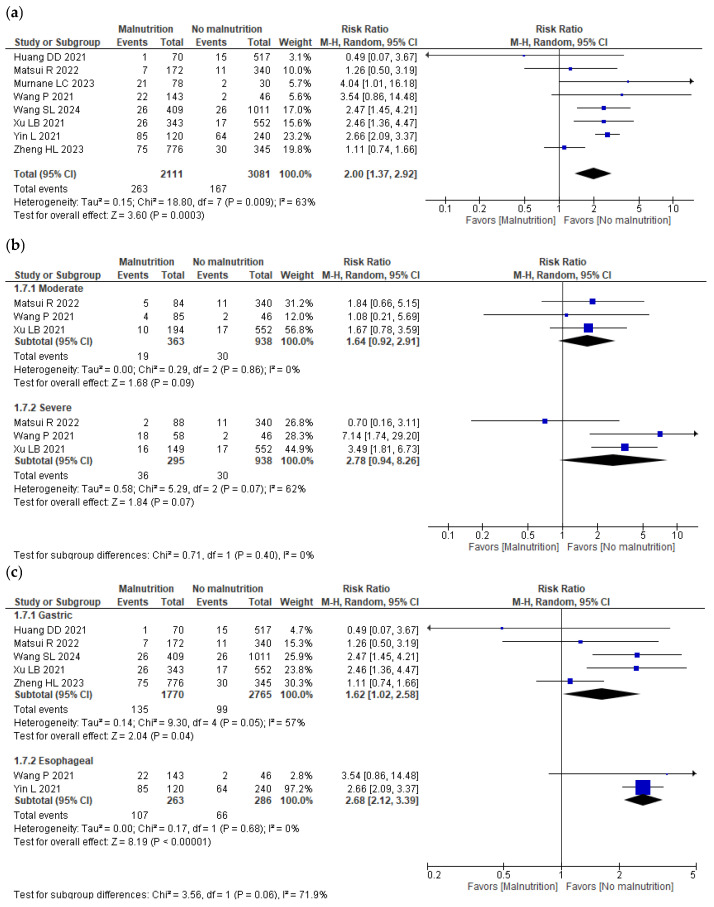

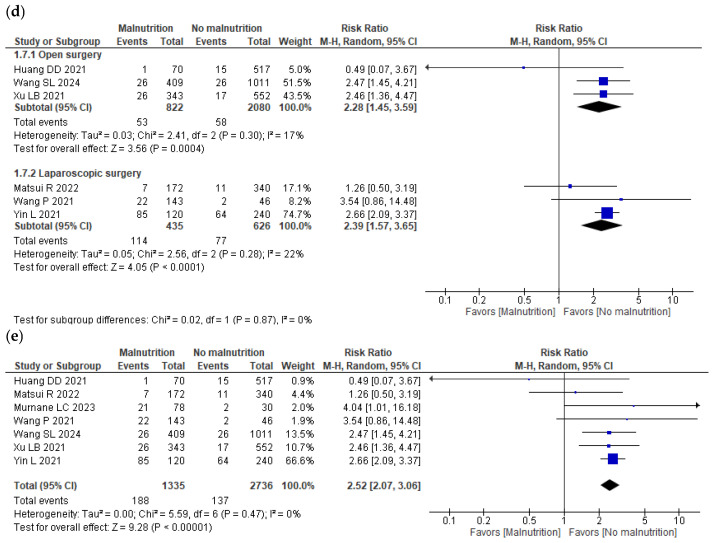
Results of meta-analyses: (**a**) postoperative pneumonia; (**b**) subgroup analysis according to malnutrition severity; (**c**) subgroup analysis by cancer type; (**d**) subgroup analysis according to surgical approach; and (**e**) sensitivity analysis excluding studies without skeletal muscle mass assessment.

Postoperative pneumonia was reported in eight studies. GLIM-defined malnutrition was associated with a higher risk of postoperative pneumonia (RR: 2.00, 95% CI: 1.37–2.92, I^2^ = 63%, n = 8, COE: low; Figure 9a). Subgroup analyses showed no clear differences in this association when stratified by malnutrition severity (Figure 9b), cancer type (Figure 9c), or surgical approach (Figure 9d), although interpretation was limited by the relatively small number of studies within each subgroup. Because of limited data, no further subgroup analyses were conducted. In a sensitivity analysis excluding studies that did not assess skeletal muscle mass, patients with GLIM-defined malnutrition continued to show an increased risk of postoperative pneumonia (RR: 2.52, 95% CI: 2.07–3.06; Figure 9e), supporting the robustness of this association.

#### 3.4.8. Mortality

**Figure 10:**
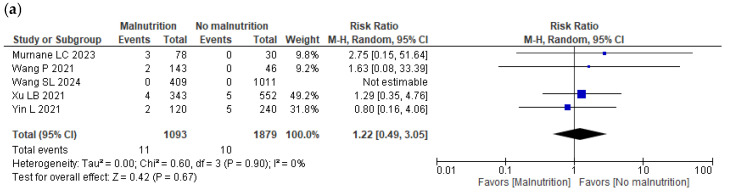

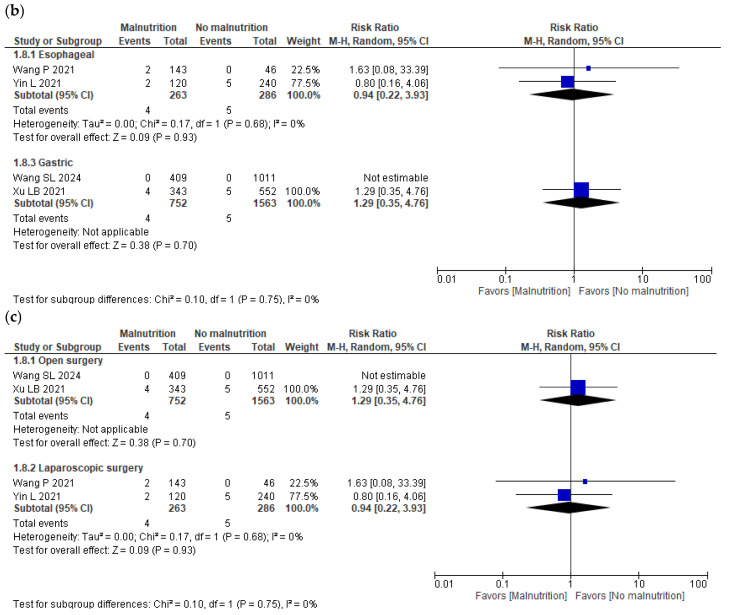
Results of meta-analyses: (**a**) postoperative mortality; (**b**) subgroup analysis in cancer type; and (**c**) subgroup analysis according to surgical approach.

Postoperative mortality was reported in five studies. GLIM-defined malnutrition was not clearly associated with an increased risk of postoperative mortality (RR: 1.22, 95% CI: 0.49–3.05, I^2^ = 0%, n = 5, COE: low; Figure 10a). Subgroup analyses by cancer type (Figure 10b) and surgical approach (Figure 10c) did not reveal meaningful between-group differences. Because only a small number of events and studies were available, no additional subgroup or sensitivity analyses were conducted for postoperative mortality.

#### 3.4.9. Postoperative Hospital Stay

**Figure 11:**
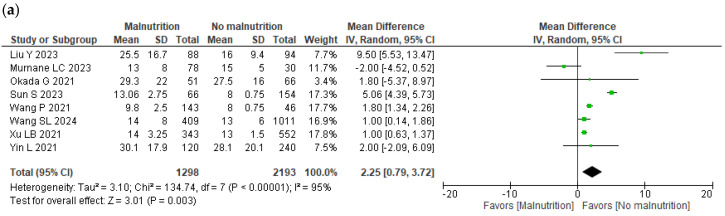

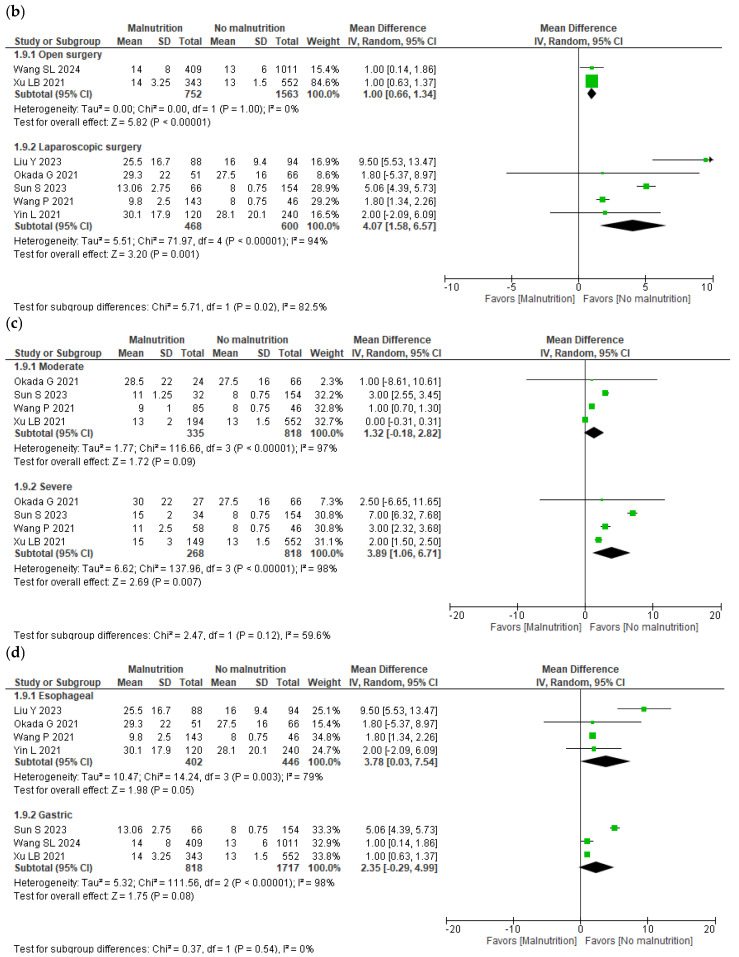

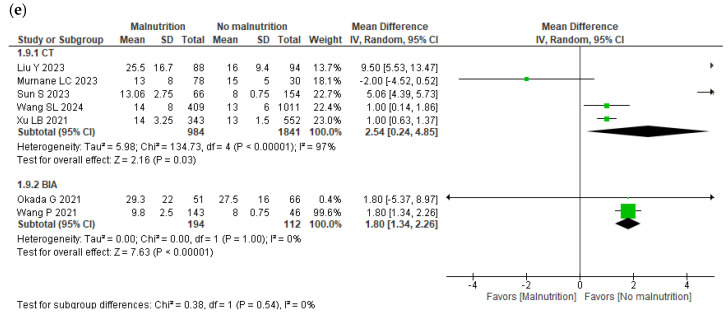
Results of meta-analyses: (**a**) postoperative hospital stay; (**b**) subgroup analysis according to surgical approach; (**c**) subgroup analysis according to malnutrition severity; (**d**) subgroup analysis in cancer types; and (**e**) subgroup analysis according to method used to assess skeletal muscle mass.

Postoperative length of hospital stay was reported in eight studies. GLIM-defined malnutrition was associated with longer postoperative hospitalization (MD: 2.25 days, 95% CI: 0.79–3.72, I^2^ = 95%, n = 16, COE: moderate; Figure 11a). In subgroup analysis by surgical approach, GLIM-defined malnutrition was linked to a significantly greater prolongation of hospital stay after laparoscopic surgery than after open surgery (P = 0.02; Figure 11b). In contrast, no clear between-group differences were observed when stratifying by malnutrition severity (Figure 11c), cancer type (Figure 11d), or method used to assess skeletal muscle mass (Figure 11e). Because of the limited amount of available data, no further subgroup or sensitivity analyses were undertaken for postoperative hospital stay.

#### 3.4.10. Publication Bias

**Figure 12:**
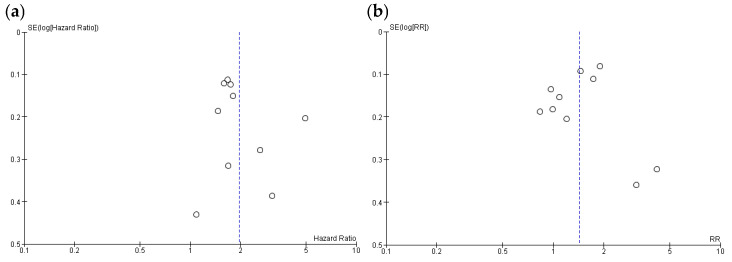
Funnel plots: (**a**) for overall survival, (**b**) for total postoperative complications.

Funnel plot inspection suggested no evident publication bias for OS (Figure 12a) or total postoperative complications (Figure 12b), as indicated by their largely symmetrical distributions. In accordance with methodological guidance [29], funnel plots were not generated for relapse-free survival or other specific postoperative complications because fewer than 10 studies were available for these outcomes.

## 4. Discussion

This systematic review and meta-analysis of 14 studies including 7876 patients suggests that GLIM-defined malnutrition may worsen OS and RFS in surgically treated patients with upper GI cancer and is associated with higher rates of postoperative complications, including overall, severe, and infectious complications, as well as anastomotic leakage and postoperative pneumonia.

In this study, GLIM-defined malnutrition was strongly associated with poorer OS in surgically treated patients with upper GI cancer, reinforcing the prognostic impact of nutritional status in this setting. Consistent with our findings, a previous meta-analysis using the Mini Nutritional Assessment reported that malnutrition was associated with a 3–8-fold increase in mortality among patients with cancer [47]. Similarly, a recent comprehensive review found that malnutrition, as assessed by several validated nutritional tools, was linked to worse long-term outcomes (HR 1.87; 95% CI, 1.62–2.17) [48]. It is important to recognize, however, that GLIM was developed as a diagnostic framework for malnutrition rather than as a stand-alone prognostic model. Applying globally endorsed, consensus-based diagnostic criteria such as GLIM will be essential for designing future clinical trials that evaluate the effects of nutritional interventions on oncologic and surgical outcomes.

In this study, GLIM-defined malnutrition was also associated with worse RFS among patients with upper GI cancer undergoing surgery. One plausible explanation is the higher incidence of postoperative complications in malnourished patients, which has been linked to poorer disease control and earlier recurrence [49]. A second key mechanism involves the impact of chemotherapy. Low muscle mass, which is a core component of GLIM-defined malnutrition, can intensify the toxicity of neoadjuvant and adjuvant chemotherapy, making it more difficult for patients to maintain dose intensity or complete planned regimens [50,51,52]. As a result, GLIM-defined malnutrition may represent an important risk factor for cancer recurrence after curative surgery. To date, only one study has specifically reported an association between GLIM-defined malnutrition and poor adherence to adjuvant chemotherapy [38], and the interplay between chemotherapy tolerance and GLIM-defined malnutrition warrants further investigation.

This comprehensive analysis indicates that GLIM-defined malnutrition is a risk factor for a broad range of postoperative complications, including overall and severe complications, infectious complications, anastomotic leakage, and postoperative pneumonia in patients with upper GI cancer undergoing surgery. Under the GLIM framework, malnutrition is defined by reduced muscle mass, significant preoperative weight loss, and low BMI, often in combination with reduced intake or inflammation, which together reflect substantial impairment of nutritional and functional reserve. Previous studies have shown that low muscle mass and preoperative body weight loss greater than 10% are associated with an increased risk of postoperative complications [4,5,6,7,8], which is consistent with our findings. Systematic reviews have also reported higher rates of postoperative pneumonia in patients with low muscle mass and BMI below 18.5 kg/m^2^, although they did not consistently find an increased risk of anastomotic leakage [7,53]. Compared with traditional sarcopenia, which focuses primarily on muscle mass and strength, GLIM-defined malnutrition incorporates inadequate nutritional intake and the presence of inflammation as additional diagnostic criteria, enabling identification of a broader spectrum of patients at risk for diverse postoperative complications rather than muscle-related outcomes alone.

Although our subgroup analyses did not show statistically significant differences between moderate and severe GLIM-defined malnutrition, the point estimates tended to be larger in the severe group across several outcomes. This pattern likely reflects limited statistical power, as only a small number of studies reported results stratified by malnutrition severity. For most endpoints, fewer than half of the included studies provided severity-specific data, which restricts the precision of pooled estimates and may underestimate the true gradient of risk according to malnutrition severity. Consequently, our meta-analysis may not fully capture the impact of progressing from moderate to severe malnutrition on postoperative and long-term outcomes. Therefore, when interpreting these results, it is important to also consider existing evidence based on non-GLIM classifications, which generally supports a dose–response relationship between the severity of malnutrition and adverse outcomes. Future observational studies and trials should routinely report outcomes by GLIM severity category to clarify these dose–response relationships and better inform risk stratification and nutritional intervention strategies.

According to our findings, a substantial proportion of patients with upper GI cancer who meet GLIM criteria for preoperative malnutrition may be candidates for targeted preoperative nutritional intervention. Reported prevalence of GLIM-defined malnutrition in the included studies ranged from 28.2% to 75.7%, with most series indicating that approximately 30–40% of patients were malnourished before surgery [30,31,32,33,34,35,36,37,38,39,40,41,42,43,44,45,46]. A recent comprehensive review reported that the GLIM criteria have good diagnostic performance for malnutrition, with a sensitivity of 0.72 and a specificity of 0.82 [54]. However, because suboptimal or inconsistent methods of assessing muscle mass can introduce heterogeneity and reduce diagnostic accuracy, skeletal muscle mass should be measured using objective, standardized indices wherever possible [54,55]. Recognizing that many undernourished patients remain undetected in routine practice, integrating structured muscle mass assessment into standard preoperative evaluation may help clinicians identify high-risk patients more reliably and initiate appropriate nutritional support before surgery.

Sensitivity analyses excluding studies without muscle mass assessment suggested that explicit evaluation of skeletal muscle mass is a stronger indicator of risk for severe complications and postoperative pneumonia than GLIM criteria applied without muscle measurements. This finding aligns with earlier meta-analyses showing that low skeletal muscle mass is associated with increased rates of major postoperative complications and pneumonia [6,7,8]. In contrast, the absence of a clear difference in long-term survival or in other postoperative complications when muscle mass was not assessed implies that some outcomes may be reasonably predicted using BMI and preoperative weight loss alone, whereas severe complications and pneumonia are better captured when muscle mass is incorporated alongside BMI and body weight loss.

Although many studies adjusted for several prognostic factors, including age, tumor stage, and comorbidities, residual and unmeasured confounding remained a major concern, as reflected by the high risk of bias in the QUIPS confounding domain. In particular, several cohorts did not fully account for neoadjuvant therapy, performance status, or comorbidity burden, which might either attenuate or exaggerate the observed associations between GLIM-defined malnutrition and postoperative outcomes. Because the number and reporting of covariates varied substantially across studies, it was not feasible to perform a robust sensitivity analysis restricted to studies with comprehensive adjustment. Therefore, the pooled effect estimates should be interpreted as potentially influenced by uncontrolled confounding in either direction.

This study has several limitations that should be considered when interpreting the findings. First, many of the included studies had a moderate to high risk of bias, which limits the overall strength and reliability of our conclusions. The overall risk of bias was driven primarily by confounding and attrition, which underpins our decision to rate the certainty of evidence for most outcomes as low to moderate according to the GRADE approach. In particular, incomplete adjustment for important confounding factors in some analyses may have led to overestimation or underestimation of the true effect sizes. Accordingly, high-quality prospective studies with robust control of prognostic variables are needed to confirm these results. Second, the small number of eligible studies restricted the depth of our subgroup analyses. In particular, analyses stratified by GLIM malnutrition severity were likely underpowered, and the available data may not fully reflect the true gradient of risk between moderate and severe malnutrition. Additional research that consistently reports outcomes by GLIM severity category is required to more accurately quantify these differences. Third, several outcomes showed substantial statistical heterogeneity. Subgroup analyses by cancer type, malnutrition severity, surgical approach, and muscle mass assessment method explained little of this heterogeneity, and we were unable to perform meta-regression because key study-level covariates were inconsistently reported. As a result, most of the heterogeneity remains unexplained, and our pooled estimates should be interpreted as average effects across clinically diverse settings rather than as stable effects applicable to all contexts. Fourth, most included studies were conducted in East Asia, particularly in China and Japan, with only one study from Australia and none from Western Europe or North America. As perioperative management, baseline BMI distributions, and nutritional practices differ between Eastern and Western settings, this geographical skew may limit the generalizability of our findings to Western populations, where complication profiles and background nutritional status can be different. Despite these limitations, this systematic review and meta-analysis is one of the first to comprehensively evaluate the impact of GLIM-defined malnutrition on both short- and long-term outcomes in surgically treated patients with upper GI cancer. Future prospective or well-designed observational studies with appropriate statistical adjustment for prognostic confounders are essential to further clarify the prognostic role of GLIM-defined malnutrition and to guide targeted nutritional interventions in this population.

## 5. Conclusions

In patients with upper GI cancer undergoing curative surgery, GLIM-defined malnutrition was consistently associated with poorer overall and relapse-free survival and with higher rates of various postoperative complications, including severe and infectious events. Although the certainty of evidence was rated as low to moderate owing to observational study designs and risk of bias, these findings reinforce the clinical relevance of systematically assessing malnutrition using a standardized consensus definition in this population. Future research should focus on prospectively validating GLIM-based risk stratification in diverse geographical settings, routinely reporting outcomes stratified by GLIM severity categories, and designing randomized or pragmatic trials that test structured preoperative nutritional interventions specifically targeting GLIM-defined malnutrition in upper GI cancer. Such studies would help determine whether using GLIM to guide nutritional support can improve postoperative outcomes beyond current practice.

## Figures and Tables

**Table 1:** GLIM diagnostic criteria.

Phenotypic criteria	Body weight loss: >5% within past 6 months or >10% beyond 6 months	Body mass index (kg/m^2^): <20.0 if <70 years or <22.0 if ≥70 years (In Asia: <18.5 if <70 years or <20.0 if ≥70 years)	Skeletal muscle mass: Below the cutoff value for each modality
Etiologic criteria	Reduced food intake or assimilation: Ingestion ≤ 50% of needs from >1 week, any reduction for >2 weeks, or any chronic gastrointestinal condition that adversely impacts food assimilation or absorption.		Inflammation: Disease burden with acute or chronic inflammation

**Table 2:** Summary of reports of included study.

Author (Year)	Country	Design	Cancer Type	Sample Size	Age (Mean)	BMI (Mean)	Male (%)	Prevalence (%) (Malnutrition)	Muscle Mass Assessment
Huang DD (2021) [30] Huang DD (2022) [31]	China	Retro	Gastric	1359	66.0	22.5	73.6	28.2%	CT
Okada G (2021) [32]	Japan	Retro	Esophageal	117	63.8	21.0	75.2	43.6%	BIA
Wang P (2021) [33]	China	Retro	Esophageal	189	65.1	22.9	68.8	75.7%	BIA
Xu LB (2021) [34]	China	Prospective	Gastric	895	65.6	22.3	74.0	38.3%	CT
Yin L (2021) [35]	China	Retro	Esophageal	360	64.1	22.4	80.8	33.3%	CC
Liu Y (2023) [36]	China	Prospective	Esophageal	182	NA	NA	79.7	48.4%	CT
Matsui R (2022) [37] Matsui R (2022) [38] Matsui R (2024) [39]	Japan	Retro	Gastric	512	67.9	22.8	65.6	33.6%	CT
Murnane LC (2023) [40]	Australia	Retro	Gastric Esophageal	108	66.4	25.9	75.0	72.2%	CT
Zhang Y (2022) [41]	China	Retro	Gastric	182	62.0	23,3	74.2	36.3%	CT
Zheng HL (2022) [42]	China	Retro	Gastric	1121	61.0	21.9	76.2	69.2%	NA
Sun S (2023) [43]	China	Retro	Gastric	220	61.8	22.2	75.5	30.0%	CT
Wang SL (2024) [44]	China	Prospective	Gastric	1420	65.5	22.5	73.6	28.8%	CT
Luo X (2024) [45]	China	Retro	Gastric	301	64.8	21.8	70.1	45.5%	CC
Zhou X (2025) [46]	China	Retro	Gastric	105	63.0	22.1	78.1	39.1%	CT

BIA bioelectrical impedance analysis; CC calf circumference; CT computed tomography; NA not applicable.

**Table 3:** Summary of findings.

Relationship Between GLIM-Defined Malnutrition and Postoperative Outcomes After Curative Resection in Patients with Upper GI Cancer				
Study Population: Adults, Exposure: With Malnutrition Defined by the GLIM Criteria, Comparison: Without Malnutrition				
Outcomes	Relative Effect (95% CI)	Patients Number (Studies)	Certainty of the Evidence (GRADE)	Comments
Overall survival	HR 1.96 (1.57 to 2.45)	5432 (10 non-RCT)	Low ^a,b^	GLIM-defined malnutrition probably worsens overall survival.
Relapse-free survival	HR 2.05 (1.45 to 2.90)	1575 (5 non-RCT)	Moderate ^a^	GLIM-defined malnutrition probably worsens relapse-free survival.
Total postoperative complications	RR 1.43 (1.14 to 1.79)	5491 (10 non-RCT)	Low ^a,b^	GLIM-defined malnutrition may increase total postoperative complications.
Severe complications	RR 2.11 (1.35 to 3.29)	3211 (8 non-RCT)	Low ^a,b^	GLIM-defined malnutrition probably increases severe complications.
Infectious complications	RR 1.39 (1.08 to 1.79)	5192 (8 non-RCT)	Moderate ^a^	GLIM-defined malnutrition probably increases infectious complications.
Anastomotic leakage	RR 1.34 (1.04 to 1.74)	5192 (8 non-RCT)	Moderate ^a^	GLIM-defined malnutrition probably increases anastomotic leakage.
Postoperative pneumonia	RR 2.00 (1.37 to 2.92)	5192 (8 non-RCT)	Low ^a,b^	GLIM-defined malnutrition may increase postoperative pneumonia.
Mortality	RR 1.22 (0.49 to 3.05)	2972 (5 non-RCT)	Low ^a,c^	GLIM-defined malnutrition may not increase mortality.
Postoperative hospital stays	MD 2.25 (0.79 to 3.72)	3491 (8 non-RCT)	Moderate ^a^	GLIM-defined malnutrition probably increases postoperative hospital stay.

CI confidence interval; GLIM global leadership initiative on malnutrition; HR hazard ratio; RCT randomized control trials; RR risk ratio. ^a^ Downgraded by one point because of moderate or high risk of bias associated with study attrition, prognostic factor measurement, study confounding, and statistical analysis. ^b^ Downgraded one point because of inconsistency due to substantial heterogeneity. ^c^ Downgraded one point because of inconsistency of forest plot.

## Data Availability

The datasets generated and/or analyzed in the current study are available from the corresponding author upon reasonable request.

## Supplementary Materials

The following supporting information can be downloaded at: https://www.mdpi.com/article/10.3390/cancers18142217/s1, Supplementary S1: PRISMA2020 checklist, Supplementary S2: The electronic database search strategy; Supplementary S3: The trial registry search strategy; Supplementary S4: Risk of bias for included studies using the Quality In Prognosis Studies tool.
